# Complete Mitochondrial Genome Sequence of the Pezizomycete *Pyronema confluens*

**DOI:** 10.1128/genomeA.00355-16

**Published:** 2016-05-12

**Authors:** Minou Nowrousian

**Affiliations:** Lehrstuhl für Allgemeine und Molekulare Botanik, Ruhr-Universität Bochum, Bochum, Germany

## Abstract

The complete mitochondrial genome of the ascomycete *Pyronema confluens* has been sequenced. The circular genome has a size of 191 kb and contains 48 protein-coding genes, 26 tRNA genes, and two rRNA genes. Of the protein-coding genes, 14 encode conserved mitochondrial proteins, and 31 encode predicted homing endonuclease genes.

## GENOME ANNOUNCEMENT

Fungal mitochondrial genomes vary greatly in size, ranging from 19 to >200 kb (1–3). They usually contain 14 genes encoding conserved proteins for oxidative phosphorylation (4); however, the number of additional genes varies greatly between species. In ascomycetes, sequenced mitochondrial genomes from the *Taphrinomycotina* and *Saccharomycotina* are <100 kb in size (5–8), whereas among the *Pezizomycotina* (filamentous ascomycetes), mitochondrial genomes can exceed 200 kb, mostly through enlargement of intronic and intergenic regions (1, 9). However, for the *Pezizomycetes*, an early diverging group of filamentous ascomycetes, no mitochondrial genomes have been described; thus, it is not clear whether the trend of genome expansion was already present in the last common ancestor of the *Pezizomycotina*. Here, the mitochondrial genome of the pezizomycete *Pyronema confluens* was sequenced to learn more about mitochondrial genome size and structure in early diverging filamentous ascomycetes.

The genomic DNA of *P. confluens* was sequenced with PacBio RS technology (BioProject no. PRJNA309361). To obtain additional mitochondrial sequences, the Illumina and 454 reads from a previous *P. confluens* genome project (10) that led to the assembly of the nuclear, but not the mitochondrial genome, were mapped to the nuclear genome with Bowtie2 (11), and nonmapping reads were extracted and the reads from bacterial contamination removed. The PacBio, Illumina, and 454 reads were assembled with SPAdes 3.6.2 (12). Among the resulting contigs, a single gapless contig of 191,189 bp contained homologs to all 14 expected conserved mitochondrial proteins. The circularity of the mitochondrial DNA was verified by PCR and Sanger sequencing of a fragment spanning the ends of the contig. Annotation was done using Prokka and MITOS (13, 14). All annotations were verified manually by BLAST analysis against GenBank (15, 16).

The genome contains genes for the conserved mitochondrial proteins for oxidative phosphorylation (*atp6*, *atp8*, *atp9*, *cob*, *cox1*, *cox2*, *cox3*, *nad1*, *nad2*, *nad3*, *nad4*, *nad4L*, *nad5*, and *nad6*), nine of which are interrupted by introns, as well as 26 tRNA genes and genes for the large and small ribosomal RNAs (*rrnL* and *rrnS*, respectively). In addition, there are 31 genes encoding putative homing endonucleases of the LAGLIDADG or GIY-YIG type, 19 of which are encoded within introns. Similar to findings in *Rhizoctonia solani* (3), truncated copies of *atp6*, *cox1*, *cox2*, *cox3*, and *nad4* reside downstream of the respective genes and are preceded by putative endonuclease genes, suggesting partial duplications of genomic regions associated with homing events. Genes for a predicted family B DNA polymerase and a reverse transcriptase often found in fungal mitochondrial plasmids (17) are also present. A gene for ribosomal protein S3 (*rps3*) was found. Similar to *Saccharomycetes*, but in contrast to many filamentous ascomycetes (18), it is not localized within an intron. In summary, the mitochondrial genome of *P. confluens* is among the largest within the fungi, indicating that a trend toward mitochondrial genome expansion was already present in the ancestor of filamentous ascomycetes.

### Nucleotide sequence accession numbers.

This mitochondrial genome project has been deposited in NCBI GenBank under the accession no. KU707476. The version described in this paper is the first version, KU707476.1.
